# Metabolic traits specific for lipid-overproducing strain of *Mucor circinelloides* WJ11 identified by genome-scale modeling approach

**DOI:** 10.7717/peerj.7015

**Published:** 2019-06-07

**Authors:** Nattapat Isarankura Na Ayudhya, Kobkul Laoteng, Yuanda Song, Asawin Meechai, Wanwipa Vongsangnak

**Affiliations:** 1 Department of Chemical Engineering, Faculty of Engineering, King Mongkut’s Institute of Technology Thonburi, Bangkok, Thailand; 2 Functional Ingredients and Food Innovation Research Group, National Center for Genetic Engineering and Biotechnology (BIOTEC), National Sciences and Technology Development Agency (NSTDA), Khong Luang, Pathum Thani, Thailand; 3 Colin Ratledge Center for Microbial Lipids, School of Agriculture Engineering and Food Sciences, Shandong University of Technology, Shandong, China; 4 Department of Zoology, Faculty of Science, Kasetsart University, Bangkok, Thailand; 5 Omics Center for Agriculture, Bioresources, Food, and Health, Faculty of Science, Kasetsart University (OmiKU), Bangkok, Thailand

**Keywords:** *Mucor circinelloides*, Lipid production, Genome-scale metabolic model, Oleaginicity, Lipid-overproducing strain

## Abstract

The genome-scale metabolic model of a lipid-overproducing strain of *Mucor circinelloides* WJ11 was developed. The model (*i*NI1159) contained 1,159 genes, 648 EC numbers, 1,537 metabolites, and 1,355 metabolic reactions, which were localized in different compartments of the cell. Using flux balance analysis (FBA), the *i*NI1159 model was validated by predicting the specific growth rate. The metabolic traits investigated by phenotypic phase plane analysis (PhPP) showed a relationship between the nutrient uptake rate, cell growth, and the triacylglycerol production rate, demonstrating the strength of the model. A putative set of metabolic reactions affecting the lipid-accumulation process was identified when the metabolic flux distributions under nitrogen-limited conditions were altered by performing fast flux variability analysis (fastFVA) and relative flux change. Comparative analysis of the metabolic models of the lipid-overproducing strain WJ11 (*i*NI1159) and the reference strain CBS277.49 (*i*WV1213) using both fastFVA and coordinate hit-and-run with rounding (CHRR) showed that the flux distributions between these two models were significantly different. Notably, a higher flux distribution through lipid metabolisms such as lanosterol, zymosterol, glycerolipid and fatty acids biosynthesis in *i*NI1159 was observed, leading to an increased lipid production when compared to *i*WV1213. In contrast, *i*WV1213 exhibited a higher flux distribution across carbohydrate and amino acid metabolisms and thus generated a high flux for biomass production. This study demonstrated that *i*NI1159 is an effective predictive tool for the pathway engineering of oleaginous strains for the production of diversified oleochemicals with industrial relevance.

## Introduction

Due to the world population growth and the industrial revolution, the value addition of agricultural materials and residues has been attributed to the sustainable production of biobased products. The prices of petroleum-derived products have gradually increased, and concern about the depletion of crude oil reservoirs has stimulated the use of alternative sources to replace the fossil-based products (Zinoviev et al., 2010; Williams et al., 2009; Dellomonaco, Fava & Gonzalez, 2010). In particular, the dedicated biobased oleochemicals and functional lipids with industrial applications (Salimon, Salih & Yousif, 2012; Vanhercke et al., 2013; Hatti-Kaul et al., 2007) are of a great interest. Oleaginous microorganisms are currently of interest as cell chassis for pathway manipulation because they exhibit phenotypes with advantages in the cultivation process and lipid production. Indeed, the particular microbes have been used in several industrial sectors, including feed, food, and biofuels (Thevenieau & Nicaud, 2013; Mata, Martins & Caetano, 2010). Of these, *Mucor circinelloides* is a well-known oleaginous fungus that accumulates lipids at high levels, particularly under certain culture conditions (Xia et al., 2011). The main storage forms in *M. circinelloides* are lipid bodies (LBs) or lipid particles (LPs), in which triacylglycerol (TAGLY) is predominant. Moreover, *M. circinelloides* is capable of synthesizing the nutritionally important polyunsaturated fatty acid (PUFA), γ-linolenic acid (GLA, C18:3 *n*-6; *cis* 6, 9, 12-octadecatrienoic acid), which has beneficial effects on human and animal health (Zurier, 1998).

The genome-scale metabolic model (GEM) is one of the computational tools used to predict metabolic behaviors and it is usually employed in systems biology in conjunction with other modern technologies, such as gene editing and synthetic biology (Thiele & Palsson, 2010). Of the oleaginous strains, the GEM of the yeast *Yarrowia lipolytica* was reconstructed, aiming to enhance the production of biodiesels and other valuable products (Pan & Hua, 2012). In oleaginous fungi, the reconstruction of the GEM of *Mortierella alpina* was implemented to investigate the metabolic characteristics for enhancing the production of lipids rich in arachidonic acid (Ye et al., 2015). Furthermore, Vongsangnak et al. (2016) also demonstrated the empowering of GEM for dissecting the growth behavior of *M. circinelloides* strain CBS 277.49 on various nutrient sources through the comparative analysis of the three GEMs of *M. circinelloides* (*i*WV1213), *M. alpina* (*i*CY1106) (Ye et al., 2015) and *Y. lipolytica* (*i*YL619_PCP) (Pan & Hua, 2012).

Among the oleaginous strains, *M. circinelloides* strain WJ11 has been identified as a promising strain for the overproduction of lipid-derived products based on its ability to accumulate lipids up to 36% of its dry cell weight (DCW) under nitrogen-deficiency conditions, which is higher than that has been reported in the reference strain CBS 277.49 (15% lipid of DCW) (Tang et al., 2017). As a consequence, the cellular metabolic mechanisms governing the oleaginicity of this particular strain have been addressed through several approaches, such as multilevel omics analysis (Tang et al., 2016; Tang et al., 2015a; Tang et al., 2015b; Tang et al., 2017). Recently, the constructed genome-scale metabolic network of *M. circinelloides* WJ11 was employed to explain its metabolic routes, focusing on lipid metabolism and carotenoid biosynthesis (Vongsangnak et al., 2018). To gain a more precise scaffold for the predictive analysis of the metabolic control involved in the oleaginicity of the WJ11 strain, a functional GEM of *M. circinelloides* WJ11 was developed in this work. Briefly, the metabolic network of *M. circinelloides* WJ11 (Vongsangnak et al., 2018) was initially used as a scaffold for improving gene annotation through metabolic reconstruction. For metabolic modeling and analysis, flux balance analysis (FBA), phenotype phase plane analysis (PhPP), fast flux variability analysis (fastFVA), and uniform sampling with coordinate hit-and-run with rounding (CHRR) were afterwards executed for observing cellular phenotypes of *M. circinelloides* WJ11. Finally, a comparison of the models between the lipid-overproducing strain WJ11 and the reference strain CBS277.49 of *M. circinelloides* was performed. This work demonstrated the efficiency and reliability of the GEM in describing growth behavior and specific metabolic traits of lipid overproduction in the *M. circinelloides* strain WJ11, which is applicable in pathway manipulation for cell optimization relevant to desired products with industrial applications.

## Materials & Methods

### GEM development of the *M. circinelloides* strain WJ11

A draft metabolic network of *M. circinelloides* WJ11 (Vongsangnak et al., 2018) was basically used for GEM development of this strain. Initially, the improved annotation of genes and relevant enzyme functions was performed using *M. circinelloides* WJ11 protein sequence homology searches for reconstructing GEM. Here, various protein and pathway databases including KEGG via BlastKOALA (Kanehisa, Sato & Morishima, 2016), EnzDP (Nguyen et al., 2015), UniProt (http://www.uniprot.org), eggNOG (Jensen et al., 2008), and JGI (genome.jgi-psf.org/Mucci2/Mucci2.home.html) were used for annotation of genes and protein functions. Concerning the expanding GEM development of *M. circinelloides* WJ11, compartmentalization information of all possible reactions was afterwards determined by a subcellular localization prediction tool, such as CELLO (Yu et al., 2006). The metabolite names and reversibility of metabolic reactions were then curated according to KEGG databases (http://www.genome.jp/kegg/pathway.html). Moreover, transport and exchange reactions were then added or deleted through network connectivity. Subsequently, GEM was converted to the form of the stoichiometric model of *M. circinelloides* WJ11. The biomass composition reaction of *M. circinelloides* WJ11 used in the model was calculated from different sources of biochemical literature and genome database. The contents of nucleotides, lipids, proteins and carbohydrates were adopted from the published research of the *M. circinelloides* strain WJ11 (Zhao et al., 2015). The compositions of amino acids and DNA were directly taken from the protein and DNA sequence information of *M. circinelloides* WJ11, respectively (Tang et al., 2015a). The compositions of lipids, carbohydrates and RNA were adopted from the published model of *M. circinelloides* reference strain CBS277.49 (*i*WV1213) (Vongsangnak et al., 2016). For energetic parameters, ATP requirements for nongrowth associated purposes (mATP) and synthesis of biomass from macromolecules (K_ATP_) and the operational P/O ratio were considered. These parameters could not be determined independently, but if one of the parameters is known the others can be estimated from experimental data. The operational P/O ratio was assumed to be 1.5 (Nielsen, 1996), the maintenance ATP (mATP) was estimated to be 1.9 mmol gDW^−1^ and the ATP requirement for biomass formation (K_ATP_) was estimated by fitting model simulations with experimental data obtained at a specific growth rate of 0.1671 h^−1^ (Zhao et al., 2015) with glucose as the carbon source. The value of K_ATP_ was hereby estimated to be 153 mmol ATP gDW^−1^.

### Model simulation and validation using FBA

FBA is a mathematical approach that is widely used for studying and identifying flux distribution through a metabolic network to generate an optimal flux towards the objective function (Orth, Thiele & Palsson, 2010). According to the steady-state assumption, the constraint-based flux simulation was performed using FBA and a linear programming solver provided by the COBRA toolbox version 3 (Heirendt et al., 2017) running through MATLAB (The Mathworks Inc., Natick, MA, USA) under the Systems Biology Markup Language (SBML). To calculate the optimal flux distribution under maximized cell growth, the biomass formation reaction was constructed and set as the objective function for model simulation under aerobic growth condition for a given substrate, such as glucose as a carbon source. For model validation, two independent experimental datasets were used (Zhao et al., 2015; Tang et al., 2016). The simulations were run by constraining the glucose uptake rate while leaving the uptake rates of nitrogen, oxygen and water unconstrained. Simulated growths were then compared with subsequent experiments.

### Characterizing metabolic phenotypes of the WJ11 strain

To characterize metabolic phenotypes of the WJ11 strain, phenotype phase plane analysis (PhPP) and fast flux variability analysis (fastFVA) were used. PhPP is a useful way to extend the study of genotype-phenotype relationships based on FBA (Bell & Palsson, 2005). This approach was used to explore sensitivity analysis describing metabolic phenotypes characteristics as a function of dual variables (Edwards, Ramakrishna & Palsson, 2002). In this study, PhPP was used to observe the growth and lipid production behaviors of this WJ11 strain as a function of glucose and nitrogen uptake rates. Briefly, two simulation conditions using the PhPP analysis were performed. The first simulation aimed to characterize the growth behavior by setting the boundaries of nitrogen (i.e., NH_3_) and glucose uptake rates in a range of 0 to 10 mmol gDW^−1^ h^−1^ and iteratively calculating the specific growth rates. In contrast, the second simulation aimed to characterize the lipid production behavior by constraining the specific growth rate and setting the boundaries of nitrogen (i.e., NH_3_) and glucose uptake rates in a range of 0 to 10 mmol gDW^−1^ h^−1^ and iteratively calculating the specific TAGLY production rates. In addition, fastFVA (Gudmundsson & Thiele, 2010) was employed to determine the flux distribution of the WJ11 metabolic network at varying nitrogen uptake rates (2–10 mmol gDW^−1^ h^−1^). At every specified nitrogen uptake rate, the flux distribution was determined as follows. First, FBA was applied to simulate the optimal flux distribution for lipid production by constraining the glucose uptake rate and growth rate at 10 mmol gDW^−1^ h ^−1^ and0.1671 h^−1^, respectively, and setting the specific TAGLY production rate as the objective function. Second, a function namely, fastFVA (Gudmundsson & Thiele, 2010), which is available in the COBRA Toolbox (Heirendt et al., 2017), was employed to calculate the possible range of fluxes for all individual reactions within the network by fixing all exchange reactions with the values obtained from FBA and then maximizing and minimizing the flux of each individual reaction. Subsequently, the relative flux change for all metabolic reactions with respect to the alteration in the TAGLY production rate was determined to identify metabolic reactions, which might be involved in lipid accumulation under nitrogen-depleted conditions. The relative flux change of a given reaction was simply calculated from the slope of a graph of the maximum flux of such a reaction in relation to the maximum flux of TAGLY production under various nitrogen uptake rates.

### Comparative GEM analysis of the lipid-overproducing and reference strains

A comparison between the models of the lipid-overproducing strain WJ11 and the reference strain CBS277.49 of *M. circinelloides* was performed. Here, the GEM of the reference strain CBS 277.49 in the form of SBML was retrieved from the previous publication by Vongsangnak et al. (2016). After that the flux distributions at optimal growth conditions of these two models were compared using fastFVA (Gudmundsson & Thiele, 2010) and CHRR (Haraldsdóttir et al., 2017) algorithms. The fastFVA yielded the minimum and/or maximum possible ranges of the fluxes whereas CHRR is a uniform sampling algorithm that provided an unbiased set of flux distributions. CHRR was also readily available in the COBRA Toolbox (Heirendt et al., 2017). Briefly, FBA was first applied to each model to calculate the optimal flux solution by setting biomass growth as the objective function. The glucose uptake rate was constrained (2.518 mmol gDW^−1^ h^−1^) (Zhao et al., 2015), while the uptake rates of nitrogen, oxygen and water were unconstrained. The next step was to fix all exchange reactions using the values obtained from FBA and then determine flux distributions using either fastFVA or CHRR. For fastFVA, once feasible range of flux distribution of each strain was obtained, the Jaccard index (Real & Vargas, 1996) was used to compare the reaction flux range between both lipid-overproducing and reference strains. The Jaccard index of an individual reaction was the ratio between the intersection and the union of the flux range in the two models. A Jaccard index of 1 indicates that both models have identical possible flux ranges, while a Jaccard index of 0 indicates that both models have completely different possible flux ranges. A Jaccard index value between 0 and 1 indicates an overlapping flux range. For CHRR, the sampling parameter values were set as 50,000 for nskips and 1,000 for nsamples for both models. The statistical *T*-test was used to compare the flux distributions between the lipid-overproducing and reference strains.

## Results

### Characteristics of the GEM of the *M. circinelloides* WJ11 strain

As shown in Table 1, the developed GEM of *the M. circinelloides* WJ11 strain (*i*NI1159) was achieved. It consisted of 1,159 genes (10.6% of total genes in *M. circinelloides* WJ11 genome), 648 EC numbers, 1,537 metabolites and 1,355 metabolic reactions, which were distributed into five compartments of the cell, namely, the mitochondria, extracellular space, cytoplasm, plasma membrane and peroxisome. It was observed that *i*NI1159 had a higher number of genes and EC numbers than *i*WV1122 due to improved gene annotation (Table 1). Noticeably, most of improved annotated genes identified in *i*NI1159 had similar EC numbers as existed in *i*WV1122, which caused a few of increased EC numbers (Table 1). The gene-protein-reaction (GPR) details of *i*NI1159 are provided in the File S1. Once compared with *i*WV1213, observably, *i*NI1159 contained 105 unique metabolic reactions distributed into carbohydrates (22 reactions), energy (18 reactions), amino acids (24 reactions), nucleotides (six reactions), lipids (13 reactions), cofactors (16 reactions), and terpenoids and polyketides (six reactions) metabolisms (File S2). Apart from the unique metabolic reactions, biomass and protein formations in *i*NI1159 were also constructed (File S3). Considering on protein formation equation, the content of amino acids, based on the amino acid sequence data of strain WJ11, had altered stoichiometric coefficients, as shown in Table 2. For metabolic connectivity, the transport and exchange reactions (122 reactions) were curated and added into the *i*NI1159 model. These transport and exchange reactions were based on the environmental sources and the product formation of the WJ11 strain. A comparative summary of the GEMs between the lipid-overproducing strain WJ11 (*i*NI1159) and reference strain CBS 277.49 (*i*WV1213) of *M. circinelloides* is shown in Table 1. The summarized biomass composition reaction of *i*NI1159 model is shown in Table 2. The full details of biomass composition can be seen in File S3. The SBML model of *i*NI1159 is also available in File S4.

**Table 1:** Metabolic characteristics of the lipid-overproducing and reference strains of *M. circinelloides*.

Characteristics	Reference strain	Lipid-overproducing strain	Lipid-overproducing strain
	CBS277.49 model	WJ11 network	WJ11 model (This study)
Name	*i*WV1213^a^	*i*WV1122^b^	*i*NI1159
Genes	1,213	1,122	1,159
EC numbers	626	640	648
Metabolites	1,413	1,278	1,537
Total metabolic reactions	1,326	1,229	1,355
Biomass formation reaction	1	–	1
Specific growth rate (h^−1^)	0.1190 (0.1192)^c^	–	0.1671 (0.1670)^c^
	0.172 (0.1889)^e^	–	0.1188 (0.1088)^d^

**Notes.**

For WJ11 model (*i*NI1159), the number of metabolites were counted in each compartment. Besides, the added/updated genes, EC numbers, and metabolic reactions in *i*NI1159 can be seen in File S1. The data in blanket are *in vivo* data.

a *i*WV1213 model was taken from Vongsangnak et al. (2016).

b *i*WV1122 network was taken from Vongsangnak et al. (2018).

c In vivo data were taken from Zhao et al. (2015).

d *In vivo* data were taken from Tang et al. (2016).

e *In vivo* data of specific growth rate for validation *i*WV1213 from Wynn et al. (2001).

**Table 2:** List of biomass composition and exchange reactions in *i*NI1159 model.

Biomass formation	153 ATP[c] + 0.44 UDPGE[c] + 0.1676 GDPFUC[c] + 0.0643 MAN[c] + 0.0018 GLYNIN[c] + 0.0026 CHIT[c] + 0.0001 CHITO[c] + 2.7618 Protein[c] + 0.1564 RNA[c] + 0.0891 DNA[c] + 0.3317 Lipid[c] ->153 ADP[c] + 153 PI[c] + BIOMASS[c]
Protein formation	5.168 ATP[c] + 0.0626 GLU[c] + 0.0591 ASP[c] + 0.0719 ALA[c] + 0.0509 GLY[c] + 0.0493 GLN[c] + 0.0494 ASN[c] + 0.0476 ARG[c] + 0.0493 PRO[c] + 0.0128 CYS[c] + 0.0834 SER[c] + 0.0623 THR[c] + 0.0255 HIS[c] + 0.0579 ILE[c] + 0.033 VAL[c] + 0.0881 LEU[c] + 0.033 TYR[c] + 0.0624 LYS[c] + 0.0249 MET[c] + 0.0111 TRP[c] + 0.0389 PHE[c] ->5.168 ADP[c] + 5.168 PI[c] + Protein[c]
Lipid formation	0.11785 ERGOSE[c] + 0.725 TAGLY[c] + 0.01955 DAGLY[c] + 0.03188 PC[c] + 0.00684 PA[c] + 0.04264 PE[c] + 0.00302 PS[c] + 0.05227 FFA[c] + 0.00093 PINS[c] ->Lipid[c]
TAGLY exchange	TAGLY[c] <=>TAGLY[e]

### Growth simulation and validation of *i*NI1159

**Figure 1:**
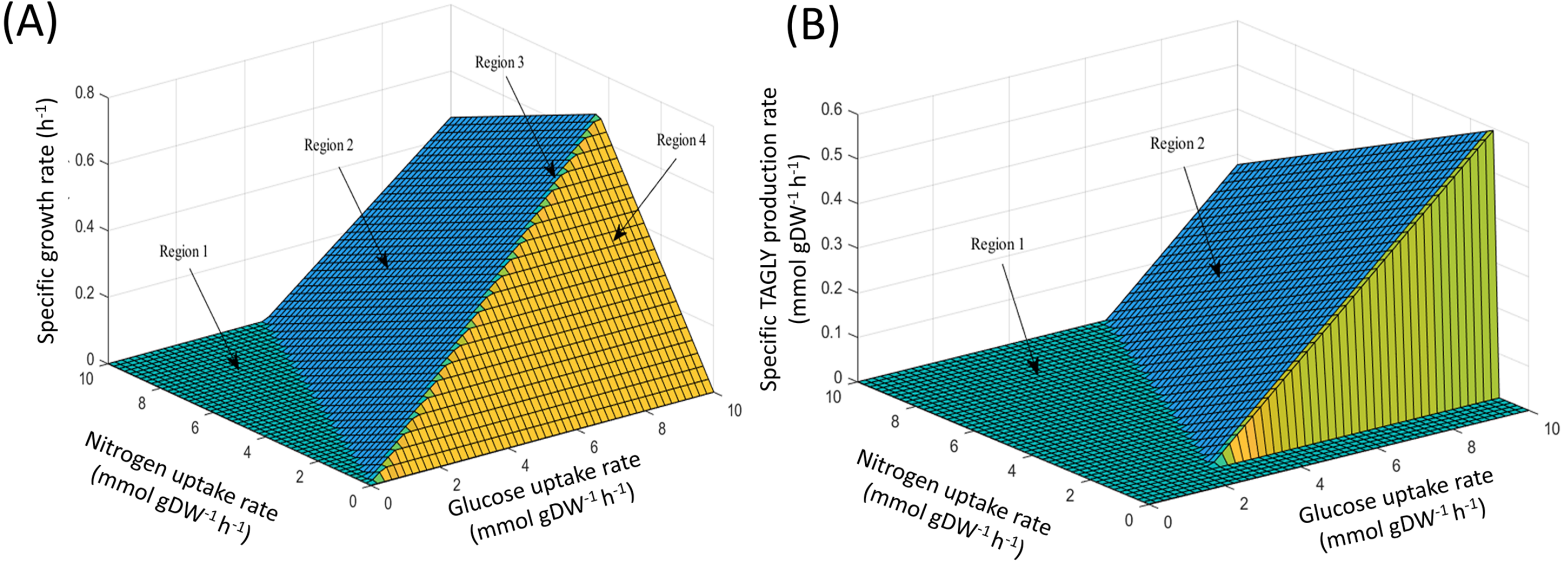
PhPP plots of metabolic phenotypic behaviors of *M. circinelloides* strain WJ11 using *i*NI1159 model. (A) Effect of glucose and nitrogen uptake rates on specific growth rate. (B) Effect of glucose and nitrogen uptake rates on specific TAGLY production rate.

Using FBA through the MATLAB environment (see ‘Materials & Methods’), the specific growth rates of *i*NI1159 on a glucose-containing medium were predicted. When the glucose uptake rates were fixed at 2.518 (Zhao et al., 2015) and 1.807 (Tang et al., 2016) mmol gDW^−^^1^ h^−^^1^ and the other environmental conditions, e.g., oxygen and nitrogen uptake rates were unconstrained, the results showed that the specific growth rates were consistent with the experimental data, in which the deviation values were 0.05% and 9.19%, respectively as shown in Table 1 (see File S5). These results suggest that the model is efficient for further analysis using the PhPP, fastFVA and CHRR approaches. The effects of nitrogen and glucose uptake rates on the cell growth and lipid production rates of *M. circinelloides* WJ11 were then investigated by PhPP analysis. As shown in Fig. 1A, the glucose uptake rate, rather than the nitrogen uptake rate, affected the biomass production rate. Although the nitrogen uptake rate was important for the biomass production rate, a low biomass growth rate was obtained under conditions with a high nitrogen uptake rate. In other words, this result suggested that the growth rate was linearly dependent on the glucose uptake rate when the nitrogen source was in excess. However, a progressive increase in the glucose uptake could not enhance the biomass growth rate if the nitrogen was limited. These results further indicated that the ratio of carbon and nitrogen contents might contribute to the growth rate. In addition, the results of the PhPP analysis indicated the effect of nitrogen and glucose uptake rates on the lipid production of WJ11 as shown in Fig. 1B. When the TAGLY production rate was set as the objective function and the biomass growth rate was fixed at 0.1671 h^−1^, the TAGLY production rate was directly proportional to the glucose uptake rate, but inversely proportional to the nitrogen uptake rate. This means that high glucose and low nitrogen conditions resulted in enhanced lipid accumulation as previously described (Tang et al., 2016). This suggests that the lipid production rate was induced by an increase in the glucose uptake rate and a decrease in the nitrogen uptake rate, which is the cultivation condition for triggering the lipid accumulation in oleaginous strains (Botham & Ratledge, 1979; Ratledge & Wynn, 2002; Ratledge, 2004).

### Major metabolic reactions associated with lipid accumulation under nitrogen-limited conditions

To identify major metabolic reactions possibly responsible for increased lipid production behavior under nitrogen-limited conditions, fastFVA followed by relative flux change analysis was applied (see ‘Materials & Methods’). Table 3 shows that the top 20 metabolic reactions with high values of relative flux change in the metabolism of carbohydrates, lipids and certain amino acids. The values of relative flux change for all metabolic reactions of *i*NI1159 when nitrogen-limited conditions are given in File S6. For carbohydrate metabolism, most of the metabolic reactions were in the glycolysis pathway, such as pyruvate oxidation catalyzed by the pyruvate dehydrogenase complex (EC: 1.2.4.1: 2.3.1.12: 1.8.1.4), and the conversion reaction catalyzed by phosphoglycerate kinase to yield D-glyceraldehyde 3-phosphate (EC: 2.7.2.3). Moreover, the flux of a metabolic reaction in the pentose phosphate pathway, which was catalyzed by ribulose-phosphate 3-epimerase (EC: 5.1.3.1) for conversion of intermediate substance from D-ribulose 5-phosphate to D-xylulose 5-phosphate, was also altered. For lipid metabolism, it was found that some reactions responsible for fatty acyl supply were markedly influent, which included the reaction catalyzed by acetyl-CoA carboxylase (EC: 6.4.1.2) for converting acetyl-CoA to malonyl-CoA, the oxidation reaction of acetoacetyl-CoA to yield acetyl-CoA catalyzed by acetoacetyl-CoA thiolase (EC: 2.3.1.9), and the reaction in biosynthesis of lanosterol to produce acetyl-CoA catalyzed by hydroxymethylglutaryl CoA synthase (EC: 2.3.3.10). Moreover, the reaction in nitrogen metabolism to convert carbon dioxide into carbonic acid catalyzed by carbonic anhydrase (EC: 4.2.1.1) was observed. Interestingly, it was detected that some amino acid reactions with relative flux change were the reaction catalyzed by threonine dehydratase to pyruvate and ammonia (EC: 4.3.1.19), the reaction catalyzed by cystathionine beta-synthase (EC: 4.2.1.22) to produce L-cystathionine, the reaction catalyzed by cystathionine beta-lyase (EC: 4.4.1.8) to generate pyruvate and ammonia, the reaction catalyzed by alanine aminotransferase to yield alanine (EC: 2.6.1.2), and the reaction responsible for converting L-serine to pyruvate and ammonia that is catalyzed by L-serine dehydratase (EC: 4.3.1.17).

**Table 3:** List of top 20 metabolic reactions with high flux change in relation to the lipid production identified in *i*NI1159 model.

**EC number**	**Metabolic reaction**	**Subsystem**	**Relative flux change**
4.1.1.1	PYR[c] ->CO2[c] + ACAL[c]	Pyruvate metabolism	21.08
1.2.4.1: 2.3.1.12: 1.8.1.4	NAD[m] + PYR[m] + COA[m] ->NADH[m] + ACCOA[m] + CO2[m]	Glycolysis	21.08
6.4.1.2	ATP[c] + HCO3[c] + ACCOA[c] ->ADP[c] + PI[c] + MALCOA[c]	Fatty acid biosynthesis	20.16
2.7.1.40	ADP[c] + PEP[c] ->ATP[c] + PYR[c]	Glycolysis	15.56
1.2.1.12	T3P1[c] + PI[c] + NAD[c] <=>13PDG[c] + NADH[c]	Glycolysis	15.56
2.7.2.3	ADP[c] + 13PDG[c] <=>ATP[c] + 3PG[c]	Glycolysis	15.56
5.4.2.1	3PG[c] <=>2PG[c]	Glycolysis	15.56
4.2.1.11	2PG[c] <=>PEP[c] + H2O[c]	Glycolysis	15.56
4.2.1.1	CO2[c] + H2O[c] ->HCO3[c] + H_PO[c]	Nitrogen metabolism	14.64
2.3.3.10	COA[c] + H3MCOA[c] <=>ACCOA[c] + AACCOA[c]	Biosynthesis of lanosterol	14.34
2.3.1.9	COA[c] + AACCOA[c] <=>2 ACCOA[c]	Fatty acid oxidation	14.34
4.3.1.19	SER[c] <=>PYR[c] + NH3[c]	Glycine, serine and threonine metabolism	11.04
4.2.1.22	SER[m] + HCYS[m] ->LLCT[m]	Glycine, serine and threonine metabolism	11.04
4.4.1.8	H2O[m] + LLCT[m] ->PYR[m] + NH3[m] + HCYS[m]	Methionine metabolism	11.04
4.1.2.13	FDP[c] <=>T3P2[c] + T3P1[c]	Glycolysis	11.04
2.6.1.2	PYR[c] + GLU[c] <=>AKG[c] + ALA[c]	Alanine/aspartate and asparagine metabolism	11.04
5.1.3.1	RL5P[c] <=>XUL5P[c]	Pentose phosphate pathway	11.04
2.7.1.11	ATP[c] + F6P[c] ->ADP[c] + FDP[c]	Glycolysis	11.04
4.3.1.17	SER[c] ->PYR[c] + NH3[c]	Cysteine metabolism	11.04
5.3.1.1	T3P2[c] <=>T3P1[c]	Glycolysis	10.04

**Figure 2:**
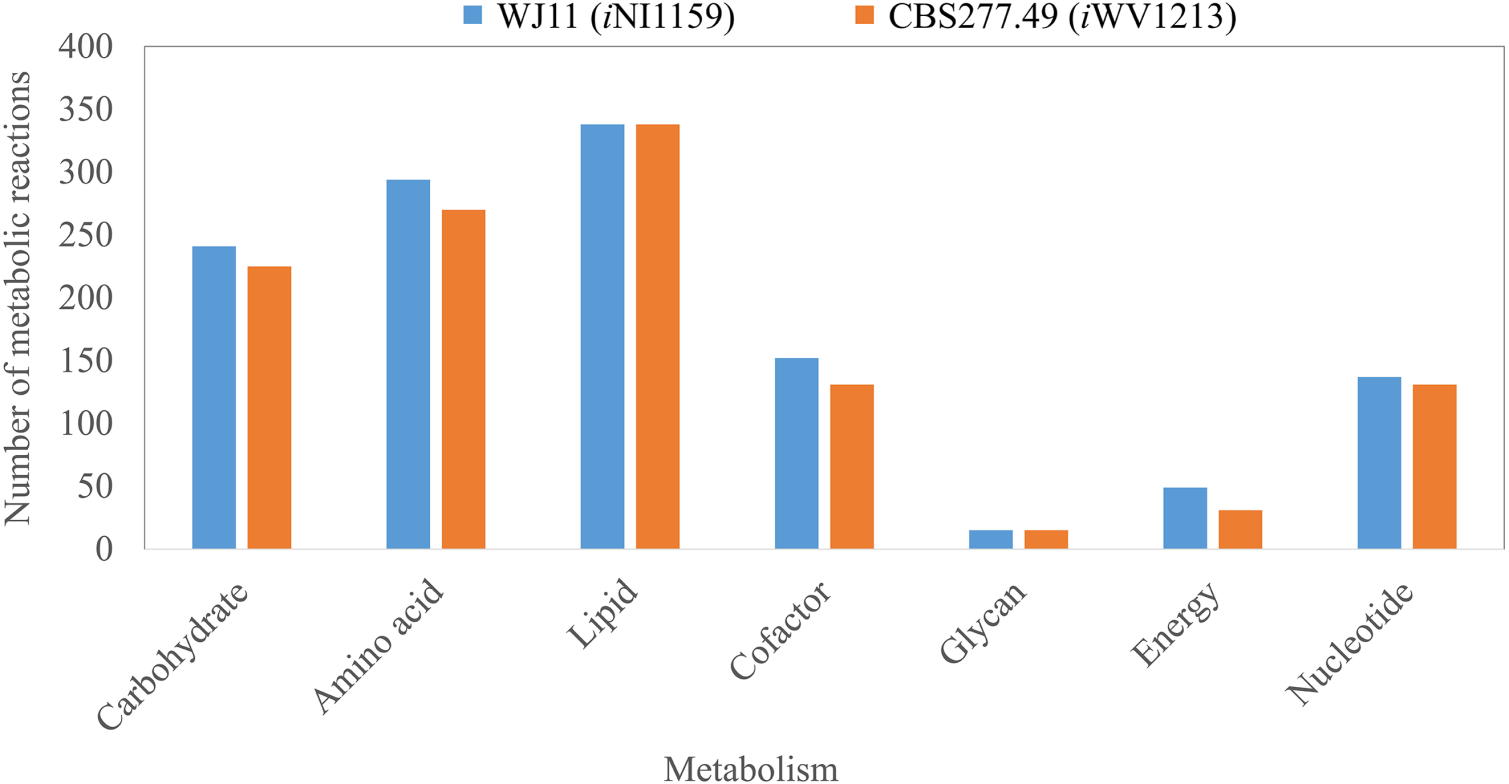
Comparison of metabolic reactions distribution between the GEMs of lipid-overproducing strain WJ11 (*i*NI1159) and reference strain CBS277.49 (*i*WV1213). Number of metabolic reactions distributing across different metabolic categories (i.e., carbohydrate, amino acid, lipid, cofactor, glycan, energy and nucleotide metabolisms).

**Figure 3:**
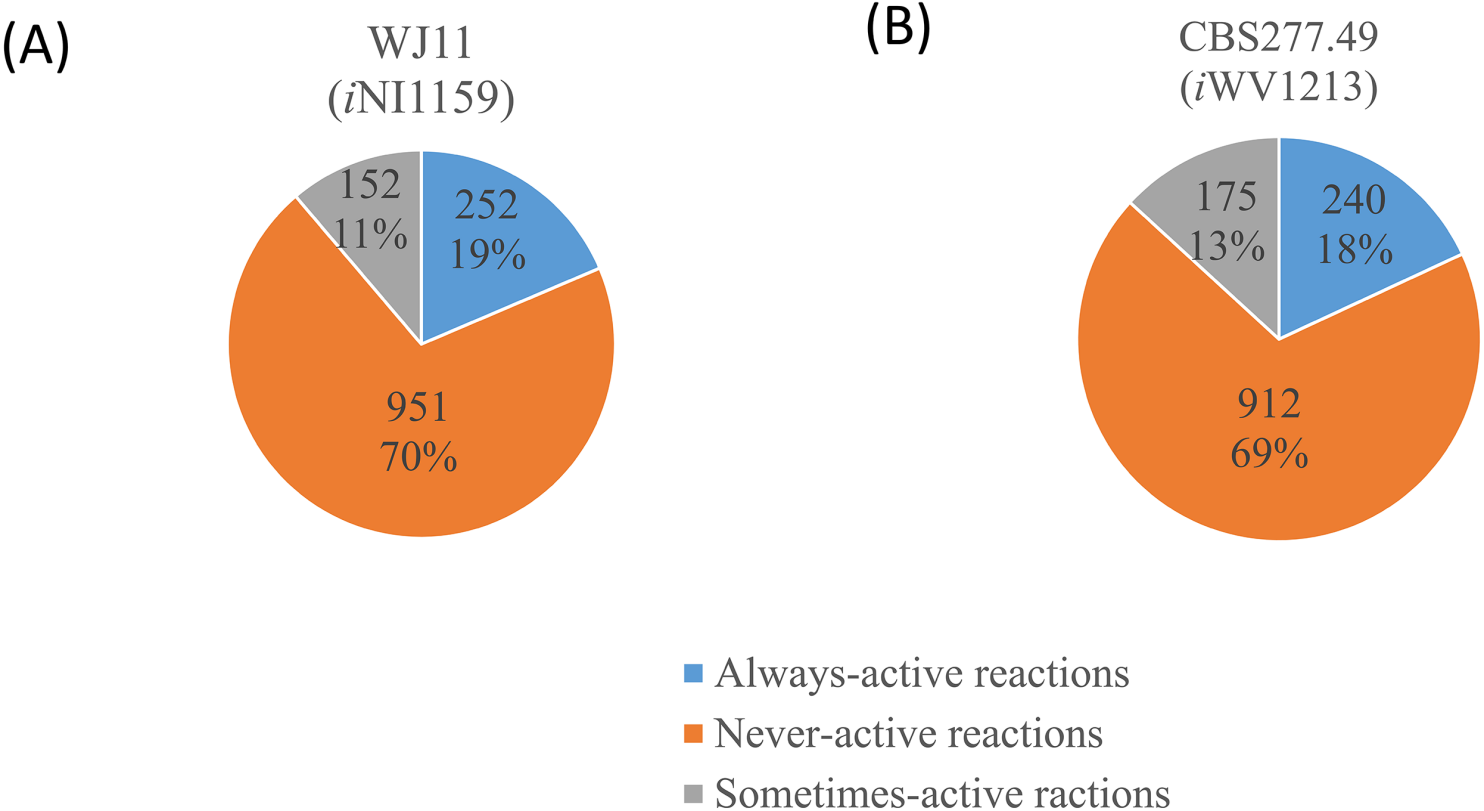
Three different categories and distributions of always-active reactions, sometimes-active reactions and never-active reactions between two *M. circinelloides* strains identified by fastFVA. (A) Lipid-overproducing strain WJ11 (*i*NI1159) and (B) reference strain CBS277.49 (*i*WV1213).

### Comparative analysis between *i*NI1159 and *i*WV1213 for guiding key metabolic routes involved in lipid accumulation

**Figure 4:**
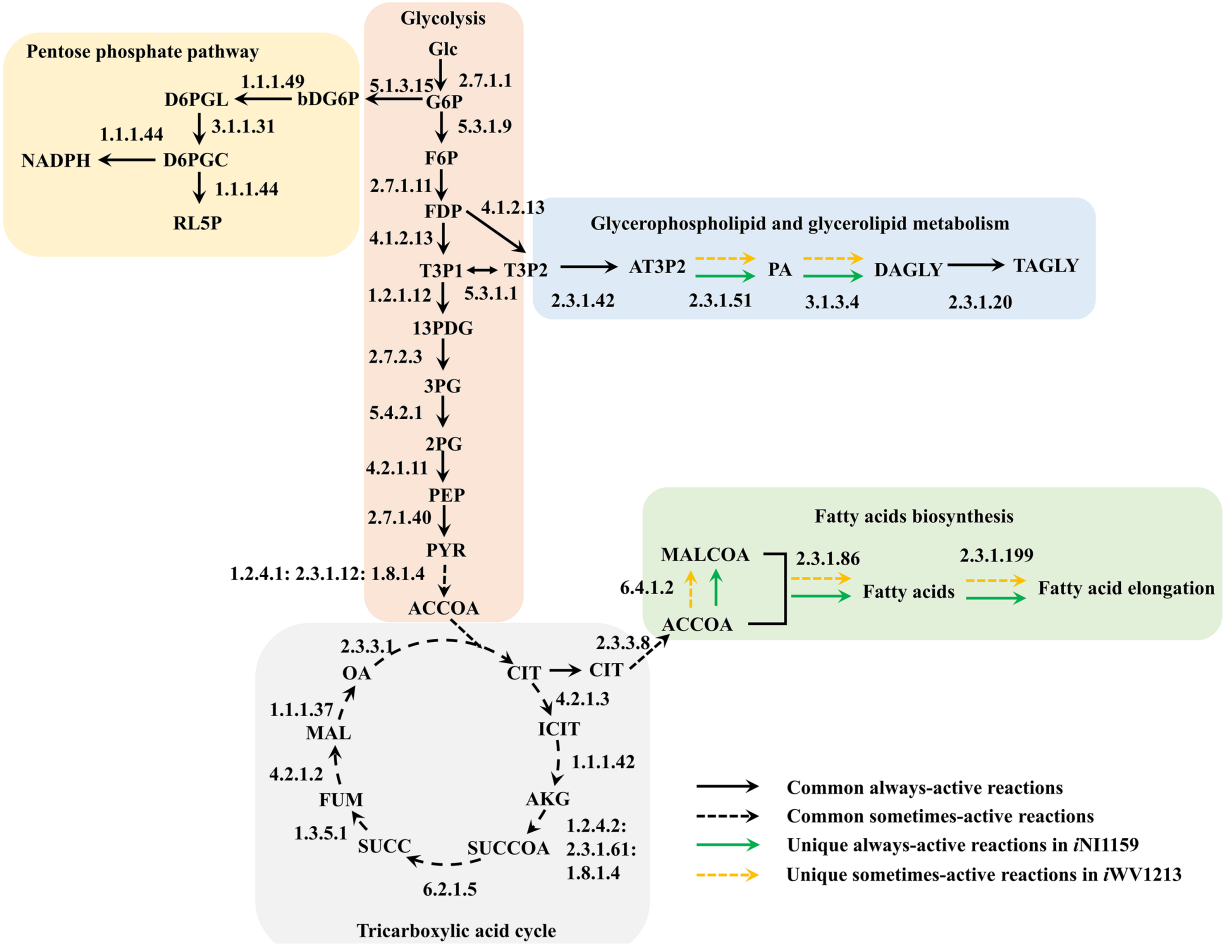
Comparative metabolic routes highlights the always-active reactions and the sometimes-active reactions between *i*NI1159 and *i*WV1213 models. The full names of metabolites can be seen in File S1.

**Figure 5:**
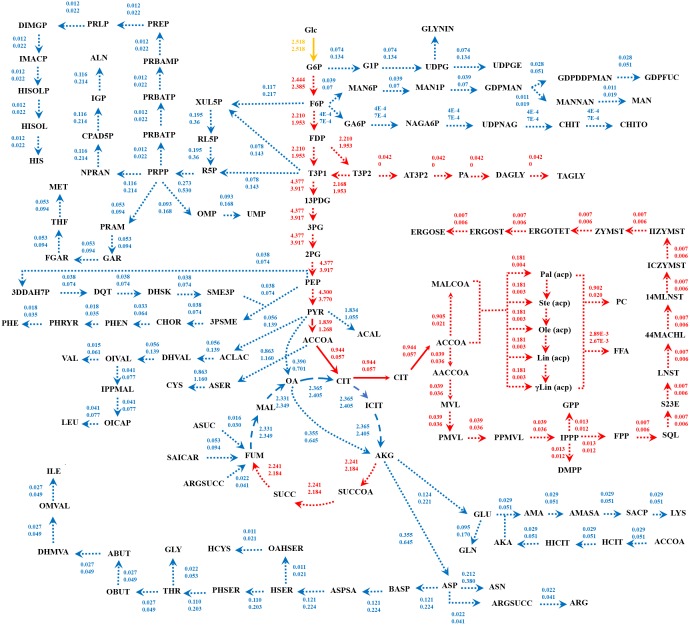
Metabolic flux distributions in central metabolic networks of *M. circinelloides* between lipid-overproducing strain WJ11 (*i*NI1159) and reference strain CBS277.49 (*i*WV1213). Fluxes of *i*NI1159 (top) and *i*WV1213 (bottom) shown are average values (mmol gDW^−1^ h^−1^) obtained from uniform random samplings. Dotted, dashed and solid arrows represent reactions with Jaccard index of 0, between 0 and 1, and 1, respectively. Reactions in red indicate higher flux flows in *i*NI1159; reactions in blue indicate higher flux flows in *i*WV1213; a reaction in yellow indicates equal flux. The full names of metabolites can be seen in File S1.

Because of the colinear evolution between the genomes of the lipid-overproducing and reference strains (Tang et al., 2015a), *i*NI1159 and *i*WV1213 undoubtedly shared almost common metabolic reactions in their metabolic networks as shown in Fig. 2. Interestingly, it was observed that the numbers of reactions in carbohydrate, amino acid and cofactor metabolisms between *i*NI1159 and *i*WV1213 were different. To gain a better understanding of the metabolic behaviors between the WJ11 and reference CBS277.49 models, the flux distributions in both models were determined and compared based on fastFVA and CHRR. Upon setting the glucose uptake rate to 2.518 mmol gDW ^−1^h^−1^ and biomass growth as the objective function, FBA was performed and showed that the specific growth rates of *i*NI1159 and *i*WV1213 were 0.1671 h ^−1^(see Table 1) and 0.2593 h^−1^ (see File S5), respectively. All solutions of the exchange reactions obtained from FBA were then fixed to perform either fastFVA or CHRR for determining flux distributions. The possible range of solutions obtained from fastFVA is shown in File S7. The fastFVA solutions were then divided into three categories, including “always-active reactions”, “sometimes-active reactions”, and “never-active reactions”. The reactions that had the minimum and maximum flux of nonzero values with the same sign were referred to as “always-active reactions”. The reactions with the minimum and maximum flux values spanning zero were called “sometimes-active reactions”. The “never-active reactions” were the reactions with both the minimum and maximum fluxes equal to zero. As a result, Fig. 3 shows that *i*NI1159 contained 252 always-active reactions (19% of total reactions), 152 sometimes-active reactions (11% of total reactions), and 951 never-active reactions (70% of total reactions). For *i*WV1213, 240 always-active reactions (18% of total reactions), 175 sometimes- active reactions (13% of total reactions), and 912 never-active reactions (69% of total reactions) were observed. Detailed information is provided in File S8 and File S9. As expected, most common always-active reactions shared between *i*NI1159 and *i*WV1213 were the reactions catalyzed by hexokinase (EC: 2.7.1.1) through pyruvate kinase (EC: 2.7.1.40), and some reactions in pentose phosphate pathway catalyzed by ribulose-phosphate 3-epimerase (EC: 5.1.3.1) through phosphogluconate dehydrogenase (EC: 1.1.1.44). The most common sometimes-active reactions were involved in the tricarboxylic acid cycle. However, the metabolic fluxes between these two strains were different, particularly in lipid metabolism, including a set of reactions catalyzed by the enzymes involved in glycerophospholipid and glycerolipid metabolism, such as lysophosphatidic acid acyltransferase (EC: 2.3.1.51), phosphatidate phosphatase (EC: 3.1.3.4), and the enzymes involved fatty acids biosynthesis (acetyl-CoA carboxylase (EC: 6.4.1.2), fatty-acyl-CoA synthase (EC: 2.3.1.86), and fatty acid elongase (EC: 2.3.1.199), as shown in Fig. 4. As a result, the *i*NI1159 had the unique always-active reactions in lipid metabolism, whereas *i*WV1213 had the unique sometimes-active reactions in lipid metabolism (File S10). Moreover, it was found that the flux distributions over the central metabolic pathways between *i*NI1159 and *i*WV1213 are different indicated by dotted arrows (Jaccard index = 0) as shown in Fig. 5. Besides, the average flux values obtained from CHRR are shown, i.e., top figures representing fluxes of *i*NI1159 and bottom figures representing fluxes of *i*WV1213 (Fig. 5). The fluxes between the two strains were found to be significantly different according to the Student’s *t*-test (File S11). It was noted that *i*NI1159 possessed higher metabolic fluxes in lipid metabolism, such as lanosterol, zymosterol, glycerolipid and fatty acids biosynthesis than *i*WV1213. In contrast, the *i*WV1213 showed higher flux distribution in the metabolism of carbohydrates (i.e., pentose phosphate pathway) and amino acids (arginine, cysteine, glycine and threonine metabolism), which were relevant to cell growth or biomass production. The detailed information of Jaccard index, average fluxes and their standard deviations as well as statistical differences (*p*-value) for the entire networks of the two models are provided in File S11.

## Discussion

GEM is an effective prediction tool by using constraint-based analysis integrated with linear programming algorithm. In this work, the developed GEM of *M. circinelloides* WJ11 expanded the metabolic information in the lipid biosynthetic pathway by incorporating the TAGLY production and exchange reaction (Table 2), which were a representative of lipids accumulated in LPs. The *i*NI1159 model could predict the simulated growth rate with low percent deviation (Table 1 and Fig. 1A). Interestingly, the lipid-accumulation stage of oleaginous strains, which is generally triggered by the carbon-excess and nitrogen-limited condition (Lamers et al., 2016), could be defined by the model (Fig. 1B). These results suggested that the developed model could be used for investigating the metabolic flux at particular conditions. As a consequence, the metabolic reactions affecting the lipid-accumulation process were identified by simulating the culture condition, in which the carbon source was fixed, and the nitrogen source was a variable or in turn the C/N ratios were varied. The top 20 metabolic reactions with high flux change in relation to the lipid production flux were found in *i*NI1159 (Table 3) when simulating the culture condition under the glucose uptake rate (2.518 mmol gDW^−1^ h^−1^) and nitrogen uptake rate (2–10 mmol gDW^−1^ h^−1^) (see File S6). Of them, some metabolic reactions have been previously identified as the most connected metabolites in the oleaginous strains, *Y. lipolytica* and *M. alpina*, such as precursors (i.e., acetyl-CoA) in lipid biosynthesis and current metabolites (i.e., ATP and NADPH) (Pan & Hua, 2012; Vongsangnak et al., 2013). Particularly, the increased fluxes of some reactions in the central carbon metabolism of *i*NI1159, such as glycolysis and pentose phosphate pathway might promote the supply of acetyl-CoA and NADPH, which are basic precursors for the lipid biosynthesis similarly to the previous findings in GEMs of the oleaginous yeast (Kerkhoven et al., 2016). These results are also coincided with the previous report of proteomic study in this oleaginous fungus, in which most enzymes in the glycolysis of *M. circinelloides* WJ11 were up-regulated under nitrogen exhaustion (Tang et al., 2016). As a result of the uniform random sampling analysis in *i* NI1159 (Fig. 5), the increased fluxes in acetyl-CoA generation of WJ11 strain by the catalytic functions of pyruvate dehydrogenase, citrate synthase, ATP:citrate lyase (ACL) and acetyl-CoA synthetase were further linked to significant increases of the fluxes toward the biosynthesis of lipids, particularly TAGLY, squalene (SQL) and steryl ester (ERGOSE or SE). These findings could explain the high lipid accumulation in *M. circinelloides* strain WJ11 by the fact that TAGLY and SE are main components of storage lipids in form of lipid body (LB) or lipid particle (LP). Recently, the efficient strategy for rational improvement of SQL production in *Y. lipolytica* by engineering the acetyl-CoA metabolism based on genome-scale metabolic model has also been reported (Huang et al., 2018).

Not surprisingly, all possible metabolic reactions in lipid metabolism, e.g., fatty acids biosynthesis and fatty acid oxidation, were among the top 20 metabolic reactions with high flux change in relation to the lipid production rate. Not only the carbohydrate and lipid metabolisms, but some reactions in amino acid metabolism appeared to contribute to lipid overproduction under nitrogen depletion. Most of them were involved in pyruvate and ammonia production (Table 3). Of them, some amino acid pathways might be targeted for genetic engineering to improve the pyruvate production, which is an important precursor for fatty acids biosynthesis. It has been reported that the redirecting carbon flux from amino acids to lipids was found in *Y. lipolytica* during nitrogen limitation by integrative analysis of multilevel omics data through the *iYali4* GEM (Kerkhoven et al., 2016).

Regarding the fastFVA, some metabolic reactions were differently displayed even though *i*NI1159 was developed using *i*WV1213 as a draft network. The common always-active reactions found in the glycolysis and pentose phosphate pathway of the two models indicated a core metabolic route for generating acetyl-CoA and NADPH, which are key precursors for fatty acids biosynthesis in common eukaryotic cells. It is noteworthy that the sometimes-active reactions in tricarboxylic acid cycle also shared among these models, which are similar to the previous findings (Kavšček et al., 2015; Vongsangnak et al., 2016). Interestingly, fastFVA indicated that fluxes in some lipid metabolic reactions categorizing either sometimes-active or always-active were different between these two models, such as glycerophospholipid and glycerolipid metabolism, as well as fatty acids biosynthesis. It could be implied that *i*NI1159 provided high lipid accumulation, whereas *i*WV1213 provided low lipid accumulation due to more sometimes-active in lipid metabolism (Fig. 4). These findings recommend that sometimes-active reactions should be further focused in more details due to these metabolic reactions may associate to both growth and lipid accumulation phases.

When compared the flux distributions between the lipid-overproducing and reference strains, both fastFVA and CHRR provided similar results. The Jaccard index based on fastFVA as well as mean fluxes obtained from CHRR indicated that *i* NI1159 had higher fluxes in the glycolysis and lipid metabolism while *i*WV1213 showed higher fluxes in the pentose phosphate, carbohydrate and amino acid metabolisms (Fig. 5). The achieved results can possibly be explained by the difference in stoichiometric coefficients of biomass equations between these two models (Table 4). Comparatively, *i*WV1213 had higher values of stoichiometric coefficients for protein leading to greater fluxes in amino acid metabolism, whereas *i*NI1159 had higher values of stoichiometric coefficients for lipid leading to greater fluxes in lipid metabolism. Notably, changes in the metabolic fluxes across amino acid metabolism of these two models were clearly observed. For examples, the metabolic fluxes of aspartate, glycine and cysteine metabolisms of *i*WV1213 were higher than those of *i*NI1159. Possibly, these might be alternative metabolic routes for leveraging between biomass and lipid production.

**Table 4:** List of different stoichiometric coefficients of biomass compositions of *M. circinelloides* WJ11 (*i*NI1159) and CBS277.49 (*i*WV1213).

Biomass composition^a^	WJ11 (*i*NI1159)	CBS277.49 (*i*WV1213)
	Stoichiometric coefficients	
Protein	2.7618	3.2950
Nucleotide		
- DNA	0.0891	0.0999
- RNA	0.1564	0.1831
Lipid	0.3317	0.1926
Carbohydrate		
- Mannose	0.064	0.075
- Fucose	0.168	0.196
- Glucuronic acid	0.440	0.515

**Notes.**

a Biomass composition of *M. circinelloides* WJ11 and CBS 277.49 at balanced growth phase (Zhao et al., 2015).

Taken together, the developed model of *i*NI1159 in this study was supported by the related experimental results, particularly in terms of physiological responses during lipid accumulation stage, where low growth rate and high lipid production rate was observed (Zhao et al., 2015; Tang et al., 2016). As a result, the *i*NI1159 model showed a tendency to produce cellular lipid at high level, whereas *i*WV1213 displayed the high biomass production (Fig. 5).

## Conclusions

The developed *i*NI1159 empowering the prediction of biomass and lipid production of *M. circinelloides* WJ11 could be exploited to explain its metabolic phenotypes through changes in metabolic flux distribution. The metabolic reactions in amino acid metabolisms influencing the lipid-accumulation process in WJ11 were identified in the nitrogen-limited conditions. The high relative flux change in carbohydrate, lipid, and amino acids metabolisms identified in the lipid-overproducing WJ11 strain by fastFVA might contribute to overflow of metabolic fluxes to lipid accumulation. Comparative metabolic flux distributions using Jaccard index and uniform random sampling clearly demonstrated distinct flux flows in the central metabolic pathways between the lipid-overproducing strain WJ11 (*i*NI1159) and reference strain CBS277.49 (*i*WV1213), enabling analysis of metabolic control involved in the oleaginicity. Thus, this *i*NI1159 model offers a promising scaffold for metabolic engineering at precise targets to achieve an optimized strain for industrial purpose.

##  Supplemental Information

10.7717/peerj.7015/supp-1 File S1 Detailed GPR information of *i*NI1159 Table S1.1: The GEM of *M. circinelloides* WJ11 (*i*NI1159).
Table S1.2: List of metabolite name, description and compartment of the *i*NI1159 model.
Table S1.3: List of improved annotated genes in the *i* NI1159 model. Table S1.4: Table S1.4: List of added/updated EC numbers in the *i*NI1159 model.

10.7717/peerj.7015/supp-2 File S2 Unique reactions identified in *M. circinelloides* strain WJ11 (*i*NI1159) and reference strain CBS277.49 (*i*WV1213) Table S2.1: The unique metabolic reactions of *M. circinelloides* (*i*NI1159).
Table S2.2: The unique metabolic reactions of *M. circinelloides* (*i*WV1213).

10.7717/peerj.7015/supp-3 File S3 Biomass compositions of *M. circinelloides* strain WJ11 (*i*NI1159) and CBS277.49 (*i*WV1213) Table S3.1: Biomass composition of *M. circinelloides* WJ11.
Table S3.2: Biomass composition of *M. circinelloides* CBS277.49 modified from (Vongsangnak et al., 2016).

10.7717/peerj.7015/supp-4 File S4 SBML model of* i* NI1159 Note: SBML model was converted using COBRA Toolbox version 3.

10.7717/peerj.7015/supp-5 File S5 FBA distributions of *i*NI1159 and *i*WV1213 Table S5.1: *i*NI1159 model simulation under glucose uptake rate of 2.518 mmol gDW ^−1^ h ^−1^
Table S5.2: *i*WV1213 model simulation under glucose uptake rate of 2.518 mmol gDW ^−1^ h ^−1^

10.7717/peerj.7015/supp-6 File S6 Relative flux change of *i*NI1159 when nitrogen-limited conditions Note: *i*NI1159 model simulation under varying nitrogen uptake fluxes (2 –10 mmol gDW ^−1^ h ^−1^) at glucose uptake rate of 10 mmol gDW ^−1^ h ^−1^.

10.7717/peerj.7015/supp-7 File S7 fastFVA distribution of *i*NI1159 and *i*WV1213 Table S7.1: fastFVA distribution of *i*NI1159 model.
Table S7.2: fastFVA distribution of *i*WV1213 model.

10.7717/peerj.7015/supp-8 File S8 fastFVA results for three types of active reactions sets in *i*NI1159 Table S8.1: fastFVA results provide always-active reactions sets.
Table S8.2: fastFVA results provide sometimes-active reactions sets.
Table S8.3: fastFVA results provide never-active reactions sets.

10.7717/peerj.7015/supp-9 File S9 fastFVA results for three types of active-reaction sets in *i*WV1213 Table S9.1: fastFVA results provide always-active reactions sets.
Table S9.2: fastFVA results provide sometimes-active reactions sets.
Table S9.3: fastFVA results provide never-active reactions sets.

10.7717/peerj.7015/supp-10 File S10 Unique always-active reactions in *i*NI1159 and unique sometimes-active reactions in *i*WV1213 Table S10.1: Unique always-active reactions in *i* NI1159.
Table S10.2: Unique sometimes-active reactions in *i* WV1213.

10.7717/peerj.7015/supp-11 File S11 Flux distributions simulated by fastFVA and CHRR, as well as Jaccard indexes and p-values of *i*NI1159 and *i*WV1213 Note:* i* NI1159 and *i*WV1213 model simulations under glucose uptake rate of 2.518 mmol gDW ^−1^ h ^−1^, and all exchange fluxes were constrained using the values at optimal growth conditions.

## Additional Information and Declarations

### Data Availability

The following information was supplied regarding data availability: The raw data and supporting information are available in the Supplemental Files.
